# A linear plasmid truncation induces unidirectional flagellar phase change in H:z66 positive *Salmonella* Typhi

**DOI:** 10.1111/j.1365-2958.2007.05995.x

**Published:** 2007-11-01

**Authors:** Stephen Baker, Kathryn Holt, Sally Whitehead, Ian Goodhead, Tim Perkins, Bruce Stocker, Jonathan Hardy, Gordon Dougan

**Affiliations:** 1 The Wellcome Trust Sanger Institute, The Wellcome Trust Genome CampusHinxton, Cambridgeshire CB10 1SA, UK.; 2 Department of Pediatrics, Stanford School of MedicineStanford, CA 94305-5402, USA.

## Abstract

The process by which bacteria regulate flagellar expression is known as phase variation and in *Salmonella enterica* this process permits the expression of one of two flagellin genes, *fliC* or *fljB*, at any one time. *Salmonella* Typhi (*S.* Typhi) is normally not capable of phase variation of flagellar antigen expression as isolates only harbour the *fliC* gene (H:d) and lacks an equivalent *fljB* locus. However, some *S.* Typhi isolates, exclusively from Indonesia, harbour an *fljB* equivalent encoded on linear plasmid, pBSSB1 that drives the expression of a novel flagellin named H:z66. H:z66+*S.* Typhi isolates were stimulated to change flagellar phase and genetically analysed for the mechanism of variation. The phase change was demonstrated to be unidirectional, reverting to expression from the resident chromosomal *fliC* gene. DNA sequencing demonstrated that pBSSB1 linear DNA was still detectable but that these derivatives had undergone deletion and were lacking *fljA*^z66^ (encoding a flagellar repressor) and *fljB*^z66^. The deletion end-point was found to involve one of the plasmid termini and a palindromic repeat sequence within *fljB*^z66^, distinct to that found at the terminus of pBSSB1. These data demonstrate that, like some *Streptomyces* linear elements, at least one of the terminal inverted repeats of pBSSB1 is non-essential, but that a palindromic repeat sequence may be necessary for replication.

## Introduction

Flagellar are complex surface-associated structures that facilitate bacterial locomotion. The extracellular flagella component that mediates propulsion of the bacterial cell is composed of a repeating protein unit known as flagellin (Macnab, 1992; 2003). Many bacteria can change the type of flagella expressed at the bacterial cell surface by alternating expression between two genes that encode antigenically dissimilar flagellins. This process, which is known as phase variation, results in antigenically distinct flagellar being exclusively expressed at the bacterial cell surface, a factor that may modify the interaction between the bacteria and the host. Flagellar may be essential for the competitive survival of some bacteria and are potent stimulator of the innate immune system through Toll-like receptor 5 (Hayashi *et al*., 2001; Feuillet *et al*., 2006).

Generally, the majority of *Salmonella enterica* serovars can mediate flagellar phase variation (Lederberg and Iino, 1956), alternating expression between two chromosomally encoded flagellin genes, *fliC* and *fljB* (Silverman and Simon, 1980). The mechanism of *Salmonella* flagellar phase variation is well described, and allows expression of only one flagellin gene at a given time (Simon *et al*., 1980). The phase change is mediated by the reversible site-specific inversion of a DNA fragment, *hin*. The *hin* region contains the promoter of the phase two flagellin gene, *fljB*. In the default orientation the promoter is facing away from *fljB*, thus the *fliC* gene is transcribed preferentially (Zieg *et al*., 1977; Zieg and Simon, 1980). Phase switching is stimulated by *hin* flipping to the opposite direction. Therefore, the promoter allows transcription of *fljB* and the adjacent downstream gene, *fljA.* The *fljA* gene encodes a protein which represses *fliC*. Monophasic *S. enterica* strains that lack the *hin*/*fljAB* locus are unable to undergo phase variation and are therefore permanently restricted to phase one.

*Salmonella* Typhi (*S.* Typhi), the causative agent of the human systemic infection known as typhoid fever (Parkhill *et al*., 2001; Parry, 2004). Typically, *S.* Typhi is an example of a monophasic *Salmonella* serovar, harbouring only the *fliC* gene (Frankel *et al*., 1989a). The *S.* Typhi *fliC* gene typically encodes the H:d (d) flagellin antigen. Some strains however, which are common in Indonesia, express a different flagellin antigen called H:j (j) (Kauffmann, 1936). The j antigen is also encoded by an allele of the *fliC* gene but the gene is truncated. A 261 bp deletion in the central region of the gene creates the j epitope, which is distinct from d (Frankel *et al*., 1989b) and may potentially alter the pathogenic potential of the bacterium (Grossman *et al*., 1995). Furthermore, Guinee *et al*. (1981) described *S.* Typhi strains from Indonesia that were motile but did not express the d or j antigen. A novel flagella antigen was discovered in these strains and named H:z66 (z66). It was observed that upon incubation with anti-z66 antiserum these strains had the ability to change phase and revert to expressing either j or d, depending on the nature of *fliC* gene on the chromosome (Guinee *et al*., 1981). These strains were therefore presumed to be biphasic and control flagella variation in a manner comparable to other *Salmonella* serovars (Moshitch *et al*., 1992).

DNA sequencing analysis of the *fljAB* region from a z66+*S.* Typhi isolate revealed similarity to the *fljAB* region of other *Salmonella*, as a putative *fljA* repressor homologue was identified downstream of the *fljB*^z66^ flagellin gene (Huang *et al*., 2004). Alignment of the amino acid sequence of the *fljAB*^z66^ region with the *fljAB* locus from *S.* Typhimurium reveals 45% homology. However, the region upstream of *fljB*^z66^ contained no inverted or direct repeats and has no DNA homology to the site-specific inversion region of *hin*.

Mobile genetic elements are known to be the largest source of genetic variation in the *S.* Typhi gene pool (Boyd *et al*., 2003) and the acquisition of novel DNA sequences have aided the specialization of this bacterial pathogen (Baker and Dougan, 2007). We have recently shown that the *fljAB*^z66^ locus is located on a 27 037 bp novel linear plasmid named pBSSB1 (Baker *et al*., 2007). The full DNA sequence of pBSSB1 has been annotated and contains no obvious repeat regions or sequences that may induce flagellar phase variation. Therefore, mechanisms responsible for changes in the expression of the *fliC* gene on the chromosome in z66+*S.* Typhi are unknown and likely to be novel. We speculatively suggest that reversion to expression of the *fliC* gene on the chromosome is catalysed by loss of the linear plasmid owing to genetic instability when exposed to a strong negative selective pressure, such as antiserum against the z66 antigen. This hypothesis is based upon previous evidence that the *fljB*^z66^ flagellin gene is undetectable by polymerase chain reaction (PCR) after initiating phase change during a laboratory assay (Huang *et al*., 2004). In this study we have found that the linear plasmid which carries the *fljB*^z66^ gene and *fljA*^z66^ is highly stable over multiple generations. Additionally, we have induced phase change in z66+*S.* Typhi strains and demonstrated that the mechanism is unidirectional and involves a deletion at the right terminus of pBSSB1.

## Results

### *fljB*^z66^ to *fliC* phase change is unidirectional

**Fig. 1:**
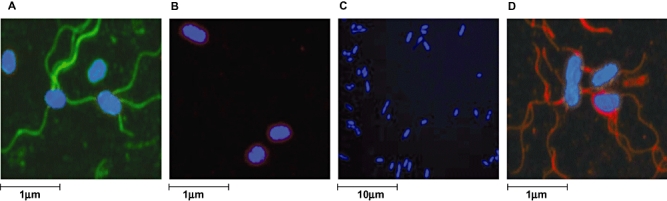
Immunoflorescence staining to visualize expression of the z66 and d *S.* Typhi flagellar antigens. A. Confocal microscopy image of 404ty*-fliC*(d) (pre-phase change) expressing the z66 flagellar antigen. The bacterial cell post incubation with anti-z66 antibody is stained blue (DAPI nuclear stain) and the z66 flagellar antigen is stained green. Scale is estimated by magnification. B. Image of 404ty*-fliC*(d) (pre-phase change) post incubation with an anti-d flagellar antibody (red) and nuclear stained with DAPI (blue). C. Confocal microscopy image of 404tya*-fliC*(d) (post-phase change). Bacterial cell is stained as A. The magnification has been decreased with respect to A to demonstrate that *S.* Typhi cells expressing the z66 antigen could not be detected after undergoing phase change. D. Image of 404tya*-fliC*(d) (post-phase change), the bacterial cell is stained as in C.

**Fig. 2:**
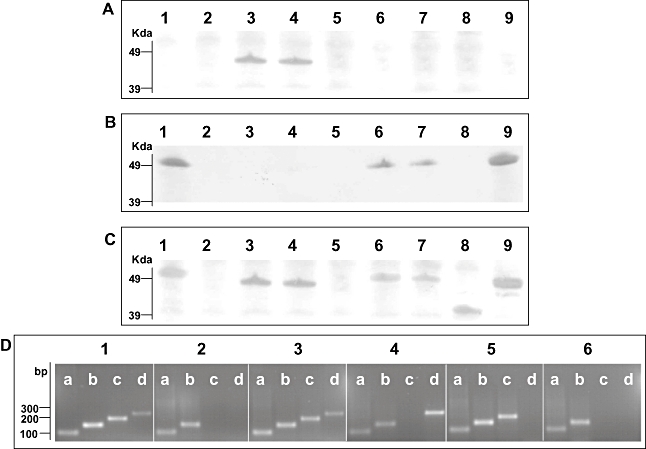
Indentification of *S.* Typhi flagellar antigens. A. Western blotting of various *S.* Typhi mid-log phase whole-cell lysates with anti-z66 flagellar antibody. Lane 1, *S.* Typhi Ty2; Lane 2, *S.* Typhi Ty2(Δ*fliC*); Lane 3, *S.* Typhi 403ty-*fliC*(j); Lane 4, *S.* Typhi 404ty*-fliC*(d); Lane 5, *S.* Typhi 404ty Δ*fljB*^z66^; Lane 6, *S.* Typhi 404ty Δ*fljA*^z66^; Lane 7, *S.* Typhi 404ty Δ*fljB*^z66^, Δ*fljA*^z66^; Lane 8, *S.* Typhi 403tya*-fliC*(j) (post-phase switch); Lane 9, *S.* Typhi 404tya*-fliC*(d) (post-phase switch). Protein sizes were estimated on SDS-PAGE gels prior to transfer with seeblue2 protein ladder (Invitrogen). B. Western blotting of various *S.* Typhi whole-cell lysates with anti-d flagellar antibody. Lanes as A. C. Western blotting of various *S.* Typhi whole-cell lysates with non-specific *Salmonella* flagellar antibody. Lanes as A. D. RT-PCR detecting mRNA transcription of *aroC* (lanes a, primers aroC_RT_F/R, 100 bp), *fliC* (lanes b, primers fliC_RT_F/R 150, bp), *fljB*^z66^ (lanes c, primers fljB_RT_F/R, 200 bp) and *fljA*^z66^ (lanes d, primers fljA_RT_F/R, 250 bp). Panel 1, genomic DNA from *S.* Typhi 404ty*-fliC*(d); panel 2, cDNA from *S.* Typhi Ty2; panel 3, cDNA from *S.* Typhi 404ty*-fliC*(d); panel 4, cDNA from *S.* Typhi 404ty*-fliC*(d)Δ*fljB*^z66^; panel 5, cDNA from *S.* Typhi 404ty*-fliC*(d) Δ*fljA*^z66^ and panel 6, cDNA from *S.* Typhi 404ty*-fliC*(d) Δ*fljB*^z66^, Δ*fljA*^z66^. Sizes are compared with the migration of Hyperladder IV (Bioline).

Two motile, agglutination positive, z66+*S.* Typhi strains (403ty and 404ty) were selected for analysis of flagellar phase variation. Both 403ty and 404ty were isolated in Indonesia and are known to harbour pBSSB1, which encodes the *fljAB*^z66^ locus. *S.* Typhi 404ty encodes a *fliC* allele that directs the expression of d [*fliC*(d)] flagellar whereas *S.* Typhi 403ty harbours a *fliC* allele encoding the j [*fliC*(j)] antigen. To examine the possibility of a mixed flagellar phase population, an aliquot of a 404ty-*fliC*(d) culture was fixed, incubated separately with anti-z66 and anti-d antiserum, fluorescently labelled and visualized using confocal microscopy (Fig. 1A and B respectively). As shown in Fig. 1A the z66 antigen (labelled green) was dominantly expressed and the d antigen (labelled red) was undetectable in the same bacterial culture (Fig. 1B). The dominance of the z66 expression was confirmed when cell lysates were analysed by Western blotting, both 403ty*-fliC*(j) and 404ty*-fliC*(d) cultures expressed the z66 antigen and the j and d antigens, respectively, were undetectable even at low levels (Fig. 2A–C, lanes 3 and 4).

*Salmonella* Typhi 403ty*-fliC*(j) and 404ty*-fliC*(d) were subjected to a number of classical phase variation assays (Collee *et al*., 1996). An inoculum of log-phase culture for each strain was introduced into a Craigie tube containing bacterial swim media supplemented with anti-z66 or control antiflagellar antiserum. As expected, both 403ty*-fliC*(j) and 404ty*-fliC*(d) generated motile colonies within 24 h of inoculation into media containing control antiserum but the appearance of motile colonies was delayed until 48 h post inoculation in media containing anti-z66 antiserum. *S.* Typhi Ty2 (d+, z66−) (Fig. 2A–C, lane 1) and *S.* Typhi Ty2 Δ*fliC* (d−, z66−) (Fig. 2A–C, lane 2) were used as control strains for each swimming assay. As predicted, the ability of Ty2 to swim in the media containing anti-z66 antiserum was unaffected and Ty2 Δ*fliC* was non-motile on prolonged incubation (data not shown).

Bacterial cells isolated from Craigie tubes containing anti-z66 antiserum inoculated with 403ty*-fliC*(j) or 404ty*-fliC*(d) were subcultured, agglutinated and subjected to further motility and phase-changing assays. All such isolated cells did not agglutinate with anti-z66 antiserum, while all such cells derived from 404ty*-fliC*(d) were motile and agglutinated with anti-d antiserum. One culture derived from 404ty*-fliC*(d), named 404tya*-fliC*(d), was stained and visualized as before, using confocal microscopy. Figure 1C shows a 404tya*-fliC*(d) culture aliquot stained with anti-z66 antiserum (stained green) and Fig. 1D shows 404tya*-fliC*(d) bacteria stained with anti-d antiserum (stained red). Post phase change, the z66 antigen is no longer detectable using antibody staining and Western blotting (Fig. 2A, lane 9) and the d antigen encoded by the *fliC* gene on the chromosome appears to dominantly expressed (Fig. 2B and C, lane 9), suggesting expression of z66 or d/j is mutually exclusive.

Bacterial cells which had changed phase and were derived from 403ty*-fliC*(j) were motile but did not agglutinate or react on Western blots with either anti-d or anti-z66 antiserum (Fig. 2A and B, lane 8). Dominant expression of the j antigen was confirmed by Western blotting with unspecific flagellin antiserum (Fig. 2C, lane 8). Cells derived from 403ty*-fliC*(j) and 404ty*-fliC*(d) were inoculated into Craigie tubes supplemented with anti-d antiserum. The motility of *S.* Typhi 403tya*-fliC*(j) was unaffected in the anti-d antiserum, whereas the z66- derivatives from 404tya*-fliC*(d) were immobilized. Bacterial cells that were derived from these immobilized 404tya*-fliC*(d) cells were subsequently cultured in swim media without antiserum and regained motility and d antigen expression.

### *fljB*^*z66*^ expression is dependant on repression of *fliC* by *fljA*^*z66*^

We have previously reported that when pBSSB2 (a derivative of pBSSB1 with a kanamycin resistance gene insertion) is transferred into a motile *Escherichia coli* isolate the recipient is rendered non-motile (Baker *et al*., 2007). However, when pBSSB2 is transferred into a motile *S.* Typhi isolate, the *S.* Typhi strain retains motility and the z66 antigen encoded by *fljB*^z66^ is preferentially expressed. These data suggest that the protein encoded by *fljA*^z66^ acts as a functional flagellin repressor in a similar mode to other FljA like regulators, thus preventing *fliC* mRNA translation (Bonifield and Hughes, 2003; Yamamoto and Kutsukake, 2006).

The putative protein encoded by *fljA*^z66^ (AM419040.1) demonstrates 98% identity in 173aa to the phase I flagellin repressor in *E. coli* AB128916.1 and 67% to the *fljA* phase I flagellin repressor (AB108532.1) of *S.* Typhimurium LT2. To study the role of *fljA*^z66^, three mutant derivatives of *S.* Typhi 404ty*-fliC*(d) were constructed, individually replacing *fljB*^z66^ and *fljA*^z66^ with a kanamycin resistance determinant (Δ*fljB*^z66^, Δ*fljA*^z66^::kan respectively) as well as generating a double Δ*fljB*^z66^, Δ*fljA*^z66^::kan mutant. All three derivatives were stable and the deletions had no obvious effect on maintenance of pBSSB2.

SDS-PAGE analysis of whole-cell lysates prepared from these mutant derivatives were transferred and probed with various antiflagellin antibodies as before (Fig. 2). 404ty*-fliC*(d) Δ*fljB*^z66^ whole-cell lysate displayed no reaction with anti-d, anti-z66 or non-specific antiflagellin antibodies (Fig. 2A–C, lane 5). The lack of apparent expression of flagellin antigen was confirmed by swimming assays, 404ty*-fliC*(d) Δ*fljB*^z66^ was non-motile in bacterial swim media (data not shown). Lysates prepared from 404ty*-fliC*(d) Δ*fljA*^z66^, in which *fljB*^z66^ is left intact, reacted with the anti-d but not the anti-z66 antibody (Fig. 2A–C, lane 6). Lysates prepared from the 404ty*-fliC*(d) *fljB*^z66^, *fljA*^z66^ mutant were indistinguishable from 404ty*-fliC*(d) Δ*fljA*^z66^, whereby they reacted with anti-d but not anti-z66 antibody (Fig. 2A–C, lane 7).

Expression of mRNA from *fliC, fljB*^z66^ and *fljA*^z66^ in the wild-type and the mutant strains was analysed using reverse transcription-PCR (RT-PCR; Fig. 2D). Expression of *fljB*^z66^ and *fljA*^z66^ was detected in mid-log phase culture in the z66+ 404ty*-fliC*(d). Additionally, expression of *fliC* mRNA was also detected in 404ty*-fliC*(d), although the d flagellin protein was not identified by Western blotting. Therefore, FljA^z66^ appears to prevent translation of the mRNA, like other *fliC* repressors (Bonifield and Hughes, 2003; Yamamoto and Kutsukake, 2006). Expression of *fliC* mRNA was also detected in Δ*fljB*^z66^, Δ*fljA*^z66^ and the double Δ*fljB*^z66^*,*Δ*fljA*^z66^ mutant. Furthermore, *fljB*^z66^ mRNA could be detected in the Δ*fljA*^z66^ strain, while z66 flagellin protein was undetected by Western blotting. These data demonstrate that if *fljA*^z66^ gene is deleted and the *fljB*^z66^ gene remains intact, the *fliC* flagellin gene is expressed dominantly and the z66 antigen is undetectable by Western blotting. Therefore, if pBSSB1 is maintained, inactivation of *fljA*^z66^ repressor, but not *fljB*^z66^, is required for reversion of expression of the *fliC* flagellin gene on the chromosome.

### pBSSB2 demonstrates a high level of stability on subculture

To investigate if loss of the linear plasmid over several hundred generations was detectable, the stability of pBSSB1 was assessed over multiple subcultures in both *S.* Typhi and *E. coli*. pBSSB1 encodes no obvious antimicrobial resistance genes, therefore pBSSB2 was again used to exploit the selectable marker. To compare the stability of pBSSB2, *E. coli* containing a variety of plasmids of assorted sizes with different antimicrobial resistance genes were selected for stability comparison, these included pUC18 (Ampcillin, 2686 bp), pBR322 (Ampcillin, 4631 bp), RSF1010 (Streptomycin, 8644 bp) and pFOS1 (Chloramphenicol, ∼40 000 bp).

**Fig. 3:**
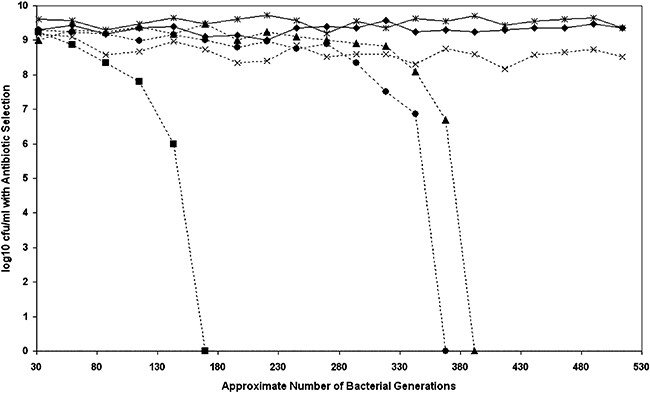
Measuring the stability of pBSSB2. Graph demonstrating the stability of pBSSB2 compared with a number of other plasmids. The approximate number of bacterial generations calculated from the initial inoculum at each subculture is represented on the *x*-axis. The log_10_ of the average cfu ml^−1^ at stationary phase for the various experimental strains calculated by serial dilution is represented on the *y*-axis. The cfu ml^−1^ was calculated by growth when diluted strains were incubated on LB media supplemented with the appropriate antibiotic. The cfu ml^−1^ was also calculated on none selective media to ensure normal culture conditions. Strains and plasmids compared; Solid line with diamond, *E. coli* pBSSB2 (kanamycin); Solid line with star, *S.* Typhi pBSSB2 (kanamycin); Broken line with cross, *E. coli* RSF1010 (streptomycin); Broken line with triangle, *E. coli* pFOS1. (chloramphenicol); Broken line with circle, *E. coli* pBR322 (ampcillin); Broken line with square, *E. coli* pUC18 (ampcillin).

Bacterial cultures were grown to stationary phase, and diluted and bacterial counts were performed after replicate plating onto none selective and appropriate selective media. The relative stability during a series of laboratory subcultures of all the plasmids over ∼500 generations is shown in Fig. 3. All the bacterial strains demonstrated equivalent stationary phase growth over the experimental period, routinely yielding between 10^8^ and 10^10^ cfu ml^−1^ on none selective media per 24 h period. The final cfu ml^−1^ per 24 h period was unchanged in all bacterial strains, this was calculated by replica plating on none selective media and was irrespective of plasmid loss (data not shown). Maintenance of the plasmids was variable but both RSF1010 and pBSSB2 exhibited the greatest level of stability. pBSSB2 demonstrated a level of stability equivalent to RSF1010, which encodes specific stabilization systems (Sakai and Komano, 1996). pBSSB2 was maintained over ∼500 generations, representing 20 subcultures without antibiotic selection in both *E. coli* and *S.* Typhi.

### Phase change is mediated by deletion of the right terminal inverted repeat

**Fig. 4:**
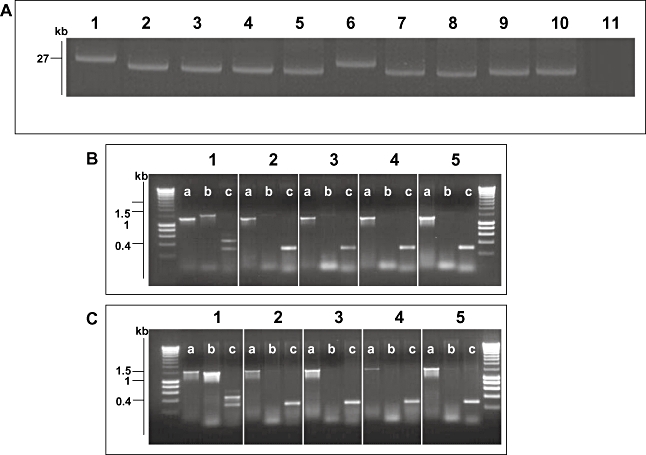
Molecular basis of *fljB*^z66^ to *fliC* phase change. A. Agarose gel electrophoresis of alkaline lysis plasmid preparation from *S.* Typhi strains pre- and post-phase switch. Lane 1, *S.* Typhi 403ty*-fliC*(j) (j); Lane 2, 403tya; Lane 3, 403tyb; Lane 4, 403tyc; Lane 5, 403tyd; Lane 6, *S.* Typhi 404ty*-fliC*(d); Lane 7, 404tya; Lane 8, 404tyb; Lane 4, 404tyc; Lane 5, 404tyd. Sizes are estimated with respect to pBSSB1 (27, 037 kbp) isolated from *S.* Typhi 403ty(j) and *S.* Typhi 404ty*-fliC*(d). B. Agarose gel of PCR amplicons produced to identify the nature of the deletion produced post phase change in strains derived from *S.* Typhi 403ty-*fliC*(j). The target for the PCR primers are; (i) *fliC*, primers fliC_F/R; (ii) *fljB*^z66^, primers z66flag_F/R; (iii) terminal inverted repeat (tir), primers tir_a, tir_d and tir_e. Each panel represents template genomic DNA from: 1, *S.* Typhi 403ty*-fliC*(j); 2, 403tya; 3, 403tyb; 4, 403tyc and 5, 403tyd. Sizes are compared with migration of Hyperladder I (Bioline). C. PCR amplifications as B using genomic template DNA from: 1, *S.* Typhi 404ty *-fliC*(d); 2, 404tya; 3, 404tyb; 4, 404tyc and 5, 404tyd.

*S.* Typhi isolates that had undergone phase change were assessed for plasmid DNA content (Fig. 4A) and evidence for the presence of plasmid DNA was found in all eight phase-changed isolates investigated. Four phase-changed derivatives from 403ty*-fliC*(j) (Fig. 4A, lanes 2–5) consistently harboured plasmid DNA ∼4 kbp smaller than wild-type pBSSB1. The same pattern was repeated in phase-changed derivatives of 404ty*-fliC*(d) (Fig. 4A, lanes 7–10). These data appear to suggest that the plasmid is stably maintained but has undergone a deletion rather than recombination or rearrangement. The resulting deletion appears to remove a fraction of the native plasmid, which presumably includes DNA sequences essential for the expression of the z66 antigen.

**Fig. 5:**
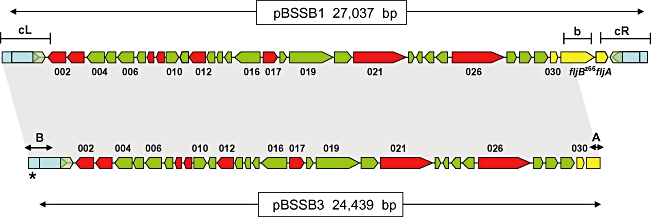
Gene map alignment of pBSSB1 and pBSSB3. The map of pBSSB1(upper) is manipulated from Baker *et al*. (2007) and depicts the 33 open reading frames encoded on the element. Predicted coding sequences with no similarities to other coding sequences in database searches are coloured green, while those with predicted functions are coloured red. Previously sequenced genes including *fljB*^z66^ and *fljA*^z66^ are coloured yellow and the tir are labelled blue. The targeted locations for primers used in Fig. 4B are demonstrated by; b, *fljB*^z66^ and the left and right tir, cL and cR respectively. The shaded region between pBSSB1 and pBSSB3 demonstrates identical sequence. The palindromic terminus sequence (as shown in Fig. 7) are labelled A and B. The asterisk distinguishes the location of the addition 120 bp at the left terminus.

Loss of the *fljB*^z66^ gene and the adjacent region was confirmed by PCR. Figure 4B and C shows PCR amplicons generated from primers targeted to *fliC*, *fljB*^z66^ and a three primer combination PCR for both terminal repeat (tir) regions from pBBSB1. Figure 4B shows PCR amplicons generated from 403ty*-fliC*(j) (1) and from phase-changed derivatives: 403ty(a–d) (2–5 respectively). Figure 4C shows PCR amplicons generated from 404ty*-fliC*(d) (1) and from phase-changed derivatives: 404ty(a–d) (2–5 respectively). All derivatives of 403ty*-fliC*(j) generated a 1295 bp *fliC* product specific for the j antigen, while all derivatives of 404ty*-fliC*(d) generated a 1495 bp *fliC* amplicon. However, sequences representative of *fljB*^z66^ were undetectable by PCR in all phase-changed derivatives, as was the PCR amplicon derived from the right tir (the upper band from 403ty*-fliC*(j) and 404ty*-fliC*(d) with primers (c). The amplicon for the left tir is indistinguishable from 403ty*-fliC*(j) and 404ty*-fliC*(d). The locations of the targets of the PCR amplifications are shown in Fig. 5. These data suggest that the unidirectional phase change is generated by deletion of specific DNA sequences from pBSSB1 which encompasses *fljB*^z66^ and *fljA*^z66^. However, owing to the linear nature of the truncated plasmids it was not possible to accurately locate the deletion points or confirm that the right tir was included in the deletion by PCR.

**Fig. 7:**
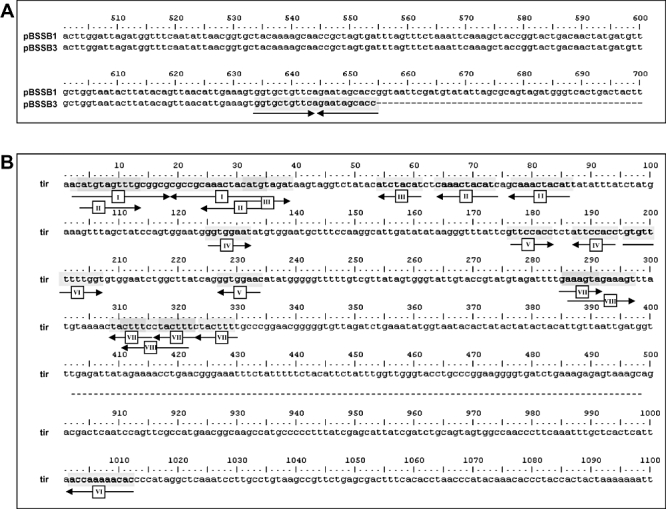
Palindromic sequences at the termini of pBSSB3. A. Sequence alignment of the *fljB*^z66^ gene from pBSSB1 and the truncated *fljB*^z66^ gene from pBSSB3. Numbers correspond to the nucleotide position within the *fljB*^z66^ gene. The palindromic sequence at the termini is highlighted; arrows correspond to the direction of the repeated sequence. B. Repeat sequence analysis of 1–500 bp and 900–1100 bp of the tir of pBSSB3, generated by blastN repeat finder. Repeat sequences (over eight nucleotides) are highlighted and numbered correspondingly. Arrows demonstrate the direction of the repeated sequence.

To precisely locate and characterize the nature of the deletion, plasmid DNA, named pBSSB3, was isolated and purified from 404tya and sequenced to completion using Illumina Solexa sequencing technology, generating a ∼×500-fold coverage of the plasmid in a single run. The deletion point of pBSSB3 in the phase-changed strain was located at the right end of pBSSB3, at position 25 439, precisely 654 bp into the *fljB*^z66^ gene (Fig. 5). *fljA*^z66^ and the right tir were undetected in the sequence data and the terminal sequence had not obviously undergone recombination with DNA sequences within the *fljB*^z66^ gene.

**Fig. 6:**
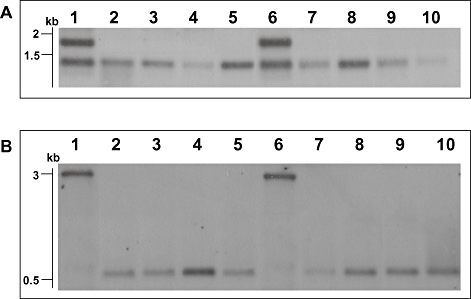
Sothern blotting investigation of truncated linear plasmid. A. Analysis of tir region of pBSSB1 and derivatives using labelled DNA prepared from a PCR amplicon (primers tir_f and tir_g) generated with template DNA from *S.* Typhi 404ty*-fliC*(d) as probe against genomic DNA digested with EcoRV. Lanes: 1, *S.* Typhi 403ty*-fliC*(j); 2, 403tya; 3, 403tyb; 4, 403tyc; 5, 403tyd; 6, *S.* Typhi 404ty*-fliC*(d); 7, 404tya; 8, 404tyb; 9, 404tyc and 10, 404tyd. Sizes estimated to migration of Hyperladder I (Bioline). B. Analysis of new right terminus of the truncated plasmids. Genomic DNA digested with SacI and probed with a PCR amplicon specific for the 5′ region of the *fljB*^z66^ gene generated with template DNA from *S.* Typhi 404ty*-fliC*(d). Lanes as Fig. 5A.

Deletion of the right tir and maintenance of the left tir was confirmed in all phase-changed strains by Southern blotting (Fig. 6A). Furthermore, the sequence demonstrated that the right end of pBSSB3 terminates at a 21 bp imperfect palindrome; GGTGCTGTTCAGAATAGCACC (Fig. 7A). The palindromic sequence in *fljB*^z66^ was confirmed to be the new terminus in 404tya and to be consistent in all phase-changed strains by Southern blotting (Fig. 6B). A sacI digestion of plasmid DNA from all strains was probed with a small PCR product from the 5′ end of the *fljB*^z66^ gene (Fig. 6B). 403ty*-fliC*(j) and 404ty*-fliC*(d) demonstrated hybridization with the probe to a ∼5 kb fragment, which is known to be the right terminus of pBSSB1. All phase-changed strains demonstrated hybridization to a 600 bp fragment, which is consistent in size with the predicted distance from the sacI endonuclease cut site to the new palindromic terminus.

The deep sequencing of pBSSB3 also generated an additional 120 bp of novel sequence which assembled on the end of the left tir. The original strain selected for sequencing of pBSSB1 was checked by PCR and the additional sequence was confirmed to be present at both termini (data not shown). Sequence analysis identified seven repeated sequences that were larger than 12 bp which may be capable of forming secondary structure within the tir. A repeat map of the tir is shown in Fig. 7B. The region contains two repeat sequences that are located in more than two locations and five repeated regions that overlap with other internal repeats (i.e. repeat sequences II and III are located within repeat sequence I and are repeated elsewhere, Fig. 7B). Most significantly, like SLP2, pSLA2 and other linear plasmids from *Streptomyces* (Hirochika *et al*., 1984; Huang *et al*., 1998; 2003; Zhang *et al*., 2006)*,* the terminus concludes at a perfect 34 bp palindromic sequence (Fig. 7B, repeat sequence I).

## Discussion

Expression of the z66 antigen appears to be default in *S*. Typhi strains containing pBSSB1, as even negligible levels of flagellin expressed from the *fliC* gene on the chromosome cannot be detected. Conversely, post phase change, incubation in appropriate flagellar antiserum renders the bacteria non-motile, they are unable to restore expression of *fljB*^z66^ and only flagellin expressed from *fliC* can be detected. Mutation of the *fljA*^z66^ gene demonstrates this is a conventional *fliC* repressor, acting post-transcriptionally and is essential for expression of the z66 antigen. Furthermore, the detection of *fljB*^z66^ mRNA but not z66 flagellin in the *fljA*^z66^ mutant suggests a natural dominance of the *fliC* gene on the chromosome or further post-transcriptional regulation. On the basis of previous data and dominance of expression of the z66 antigen we hypothesized that phase change may be catalysed by loss of pBSSB1 when subjected to a negative selective pressure. The loss of pBSSB1 would ultimately be responsible for the deletion of *fljB*^z66^ and *fljA*^z66^, which are essential for expression of the z66 antigen (Huang *et al*., 2004). However, post phase change, all examined strains maintained a truncated pBSSB1 derivative. A high level of plasmid stability after the phase-changing assay correlated with the stability of the linear element over a prolonged period of subculture. pBSSB2 was consistently maintained without selection over 500+ generations in both *Salmonella* and *E. coli*.

Reversion to expression of *fliC* was confirmed to be unidirectional and involves a deletion at the right end pBSSSB1. The deletion may be stimulated by a replication error at the novel terminal palindrome and encompasses *fljA*^z66^, the right tir and a large proportion of the *fljB*^z66^ gene. As in other organisms harbouring a linear DNA element, rearrangements and deletions are often encountered at the termini and are responsible for the substantial level of variability observed in these regions (Birch *et al*., 1990; Fischer *et al*., 1998; Volff and Altenbuchner, 1998). In common with some linear elements from *Streptomyces*, both tir of pBSSB1 are not required and one may be removed without any detrimental effects on replication (Rauland *et al*., 1995). Furthermore, a large amount of genetic information adjacent to the telomeres in *Streptomyces* linear chromosomes has been shown to be superfluous under laboratory conditions (Redenbach *et al*., 1993; Wenner *et al*., 2003). Generally, genes essential for maintenance are located in a region of higher stability in close proximity to the origin of replication, ensuring survival (Bentley *et al*., 2002). An additional example of instability may lie at the left hand end of pBSSB1, coding sequences 002, 003 and 004 are all putative methyltranferases and 003 and 004 share 59% and 36% amino acid homology, respectively, with 002. Conservation between these three simultaneous paralogues may suggest a historic amplification event of the original gene adjacent to the tir.

We have previously demonstrated that the 5′ end of pBSSB1 DNA is insensitive to 5′-3′ exonuclease treatment (Baker *et al*., 2007). The mechanism for replication used by many *Streptomyces* linear plasmids involves terminal proteins bound to the 5′ of the DNA via secondary structure in the DNA, which is formed by palindromic repeat sequence (Bao and Cohen, 2001, 2003). Additional DNA sequence at the termini demonstrated that pBSSB1 contains several repeat sequences with the extremities of the tir and terminates at a 34 bp palindrome. However, the tir and the palindromic DNA sequence within the tir on pBSSB1 display no similarity to previously described sequences from any bacterial species known to harbour linear DNA complexes. Nonetheless, evidence presented here adds some insight into the biology of this element and strengthens our hypothesis that pBSSB1 replication is similar to many *Streptomyces* linear plasmids. Replication of such elements is known to occur bi-directionally from a central origin and a terminal 5′ protein binds to the palindromic DNA (Chang and Cohen, 1994; Qin and Cohen, 1998). Therefore, although the precise mechanism of replication requires elucidation we suggest pBSSB3 retains the ability to replicate via this replacement palindromic sequence within the *fljB*^z66^ gene.

Despite extensive screening of worldwide *S.* Typhi collections, data demonstrate that z66+ strains have only ever been isolated in Indonesia or from travellers returning from Indonesia (Vieu *et al*., 1986; Tamura *et al*., 1988; Vieu and Leherissey, 1988). It has been proposed that z66+*S.* Typhi strains with the ability to switch phase are the ancestors of global *S.* Typhi (Moshitch *et al*., 1992). Whereby, *S.* Typhi became established as a specialized pathogen of man in Indonesia and *S.* Typhi from other parts of the world descended from an Indonesian monophasic d subclone. However, recent analysis of single nucleotide base changes located around the chromosome of *S.* Typhi has demonstrated that z66+ strains may represent an isolated haplotype circulating only within Indonesia (Roumagnac *et al*., 2006). Indonesia has a high incidence of typhoid fever (Vollaard *et al*., 2004; 2005) and evidence implies there may be immune selection occurring within the Indonesian population. Therefore, we suggest that selective pressure sustains strains harbouring pBSSB1 only within this endemic typhoid region. Additionally, z66+ strains prevented from radiating to other countries in the locality, which is uncommon in the nature of spread of *S.* Typhi (Roumagnac *et al*., 2006). The data presented here establish that z66+*S.* Typhi strains cannot undergo ‘true’ phase variation like other *Salmonellae* and appear, in essence, to be monophasic. Theoretically, *S.* Typhi strains that have undergone phase change could re-express the z66 antigen, which would rely on re-acquisition of the native plasmid in the environment. The promiscuity of such linear elements is unknown, and currently we are unaware if reversion to expression of *fliC* and maintenance of the linear plasmid occurs outside laboratory conditions, which is difficult to predict an advantage of a unidirectional phase change in the natural environment of the bacteria. It is possible that other genes with unknown function that are located on pBSSB1 may also confer a selective advantage to the bacterial cell or the partitioning mechanism is extremely efficient and permits an entirely parasitic existence for this element.

pBSSB1 and its derivatives appear to be an adept selfish mobile genetic element. The acquisition of a functional flagellar and flagellar repressor gene has ensured continued existence of this linear plasmid within the *S.* Typhi population in Indonesia. Possession of accessory genes that are unessential for replication on such mobile elements aid maintenance, such as antibiotic resistance genes and heavy metal resistance genes found on many plasmids. The nature of the relationship between chromosomal and extra-chromosomal DNA appears to be symbiotic, whereby possession of the mobile genetic element aids the bacterial life style in the niche in which it circulates.

## Experimental procedures

### Bacterial strains

All bacterial strains utilized in this study are from the The Wellcome Trust Sanger Institute collection. Phase-changing assays were performed using two z66+ strains originally isolated in Indonesia; *S.* Typhi 403ty*-fliC*(j) and *S.* Typhi 404ty*-fliC*(d). Individual bacterial colonies were selected from a number of different assays post phase change, these were named *S.* Typhi 403tya, b, c, d and *S.* Typhi 404tya, b, c, d and were isolated from initial inoculums of *S.* Typhi 403ty*-fliC*(j) and *S.* Typhi 404ty*-fliC*(d) respectively. Control strains were *S.* Typhi Ty2 and a *fliC* mutant constructed in *S.* Typhi Ty2 (see below). Three mutant strains (*fljB*^z66^::kan, *fljA*^z66^::kan and *fljB*^z66^*fljA*^z66^::kan) were constructed (see below) in strain *S.* Typhi SGB31 containing pKD46 and pBSSB1 (Baker *et al*., 2007). Stability assays were performed using *S.* Typhi and *E. coli* strains containing pBSSB2, SGB34 and SGB33 respectively (Baker *et al*., 2007), and *E. coli* top 10 cells (Invitrogen) transformed with pUC18, RSF1010, pBR322 and pFOS1.

### Phase change

Flagellar phase-changing assays were performed as outlined previously (Collee *et al*., 1996). Briefly, Craigie tubes were prepared using sterilized plastic bijou vessels containing 3 cm of Tygon R-1000 tubing exposed slightly from the media. The tubes contained 4 ml of bacterial swim media (0.6% Luria–Bertani (LB) agar and LB broth with 16% gelatine, autoclaved and mixed 1:1) with no antiserum or supplemented with 1/500 dilution of either; rabbit anti-z66, mouse anti-d (Bio-stat, UK), mouse anti-*Salmonella* flagellar (Bio-stat, UK) antiserum. 500 μl of mid-log growth phase (0.6 OD_600_) *S.* Typhi cells were inoculated into the tubing and incubated without agitation at 37°C until motile bacteria were observed on the exterior of the tubing and/or the surface of the media.

### Immunofluorescence

*S.* Typhi cells expressing flagellar were prepared for immunofluorescence imaging using a method used to visualize other *S.* Typhi surface antigens (Wain *et al*., 2005). After fixing and washing the bacterial cells were incubated in either rabbit anti-z66 or mouse anti-d antiserum (as above). Following several washes the fixed cells were incubated in an appropriate fluorescent secondary antibody anti-rabbit/mouse IgG (Alexoflor) and DAPI nuclear stain to identify the bacterial cells. Slides were visualized and photographed using a Zeiss Axiovert 200 M microscope fitted with 25 M 510 Laser module.

### Plasmid stability

The stability of pBSSB2 was calculated in *S.* Typhi and *E. coli* in comparison to a number of other plasmids transferred by electrotransformation into *E. coli* top 10 (Invitrogen). Bacterial cells were initially grown overnight with agitation at 37°C in 10 ml LB broth supplemented with the appropriate antibiotic to ensure plasmid selection. Cells were enumerated by serial dilution and plating onto none selective and selective LB agar plates. After initial culturing and for the course of the experiment, approximately 100 cfu of stationary phase bacterial were inoculated into 10 ml of LB broth without selection, every 24 h period. The stability of the various plasmids and pBSSB2 was assessed by serial dilution in PBS and comparative enumeration after plating onto selective and none selective LB agar plates. The precise inoculums were counted daily and the approximate number of bacterial generations was established by calculating the number cell divisions required to generate the final stationary growth phase cfu ml^−1^.

### Mutagenesis

All *S.* Typhi mutants were constructed using a modified version of the lambda red recombinase (one-step method) described by Datsenko and Wanner (2000). PCR products were amplified from pKD3 or pKD4 template DNA in 10 50 μl of reactions with the following primers; *fliC* mutation, H1P1 cgagcgtttgtcttccggtctgcgtatcaacagcggtgtaggctggagctgcttc and H2P2 cggttgcgtagtcg gaatcttcgatacggctacggcatatgaatatcctccttag; *fljA*^z66^ mutation, rep_del_F cggggcttttttgtttaatgtgctaaaactggt tctaaggtacaactgtttgtgtaggctggagctgcttcg and rep_del_R tctggaatatccttttgaaaatattgataaccaactgataa tatgaaggtcatatgaatatcctcctta; *fljB*^z66^ mutation, flag_del_F cttgaaagacacaggtaagcctgacttatataccca aaaggaaaatatttgtgtaggctggagctgcttcg and flag_del_R cattaaacaaaaaagccccgcaatcacggggcttagtactata aactatacatatgaatatcctcctta; double *fljB*^z66^*fljA*^z66^ mutation flag_del_F and rep_del_R. PCR amplified DNA was pooled, precipitated and re-suspended in 10 μl of nuclease-free water. Re-suspended DNA was mixed with 50 μl of competent *S.* Typhi SGB32 cells (Baker *et al*., 2007) or *S.* Typhi Ty2 cells containing pKD46 (grown in LB broth, supplemented with 0.1 M arabinose, and harvested at 0.3 OD_600_) in 2-mm electroporation cuvettes (Invitrogen). Cells were electrotransformed (2.4 kV, 600 ohms, 25 lF; Bio-Rad Gene Pulser, http://www.bio-rad.com), allowed to recover for 2 h statically at 37°C in 400 μl of SOC, and then plated onto LB medium supplemented with 25 μg ml^−1^ chloramphenicol or kanamycin. pKD46 was destroyed by heat treatment as described and correct insertions were checked by PCR.

### Western blotting

Mid-log phase (0.6 OD_600_) *S.* Typhi cells were harvested, resuspended in PBS and boiled for 10 min in 1× SDS-loading buffer. Twenty microlitres of the subsequent whole-cell lysate solution was loaded onto a SDS NU-Page 4–12% bis-tris gel (Invitrogen) and electrophorized for 1 h at 150 V in NU-Page MES SDS running buffer (Invitrogen). Protein was transferred onto protran nitrocellulose paper (Whatman), using semi-day blotting apparatus (Bio-Rad) 15 V for 1 h. Blots were incubated in 1/1000 dilution of appropriate antiflagellar antiserum (as above), washed and imaged on the membrane using the opti-4CN detection reagents (Bio-Rad) as described by the manufacturer.

### Southern blotting

Southern blotting was carried out using Hybond N+ nitrocellulose (Amersham Lifesciences). Probes were prepared from purified PCR products (PCR purification kit; Qiagen, http://www.qiagen.com) amplified from *S.* Typhi 404ty*-fliC*(d) genomic DNA. Primers tir_f and tir_g generated a PCR amplicon for the tir, as previously described (Baker *et al*., 2007) and primers; fljB_probe_F ccgctatcgagcgtctgtcttc and fljB_probe_R tgaacagcaccactttcaatg generated an amplicon for the 5′ of the *fljB*^z66^ gene. Purified PCR products were labelled using the Gene Images CDP-Star and AlkPhos Direct Labelling kit (GE Healthcare, http://www.gehealthcare.com). Detection was performed with the Gene Images CDP-Star Detection kit. The sizes of restriction fragments were estimated by comparing migration distances against Hyperladder I (Bioline, http://www.bioline.com).

### Plasmid isolation

Plasmid DNA was prepared using an alkaline lysis method originally described by Kado and Liu (1981). The resulting plasmid DNA was separated by electrophoresis in 0.7% agarose gels made with 1× E buffer. Gels were run at 90 V for 3 h, stained with ethidium bromide and photographed.

### PCR and RT-PCR

Polymerase chain reaction (Fig. 4B and C) was performed using purified genomic DNA (wizard genomic DNA extraction kit, promega) as template DNA using primers specific for *fliC* (fliC_F and fliC_R), *fljB*^z66^ (z66flag_F/R) and tir (tir_a, tir_d and tir_e) as previously described (Baker *et al*., 2007). PCR was performed in a 25 μl volume using PCR supermix Taq polymerase (Invitrogen) and cycled on an MJ research thermal cycler under the following conditions; 94°C for 30s ×1 (94°C for 30 s, 55°C for 30 s, 72°C for 2 min ×30) and 72°C for 4 min.

RNA was purified with RNeasy RNA extraction kit (Qiagen) from mid-log phase *S.* Typhi cells treated with RNA protect (Qiagen) as recommended by the manufacturer. Quality and quantity of purified RNA was examined on a ND-1000 spectrophotometer (Nanodrop). The recommended amount of RNA was DNase treated and tested by PCR to ensure efficient hydrolysis of the DNA before generation of cDNA using superscript II reverse transcriptase (Invitrogen). cDNA was amplified with primers specific for *aroC* (aroC_RT_F tctgcgatcggcactgcgcg and aroC_RT_R tgccgggacgtacgatcaac), *fliC* (fliC_RT_F taacgcagtaaagagaggac and fliC_RT_R cctgtcttctgcccgtagcc), *fljB*^z66^ (fljB_RT_F atggcacaagtcatcaatac and fljB_RT_R ttacgggacgcctgagtcag) and *fljB*^z66^ (fljA_RT_F atgaatgatatctcatatgg and fljA_RT_R gcaattcttcgagtgatgcg). PCR was performed as described above with the exception of the cycle; 94°C for 30 s ×1 (94°C for 30 s, 50°C for 30 s, 72°C for 1 min ×30) and 72°C for 2 min PCR products were examined on a 2% agarose TAE gel.

### DNA sequencing and analysis

Plasmid DNA from strain *S.* Typhi 404tya was isolated and named pBSSB3. pBSSB3 DNA was sequenced using Solexa, Illumina genome analyser technology (Bennett, 2004; Mikkelsen *et al*., 2007). The assembly of the DNA was performed using SSAKE (Warren *et al*., 2007) and repeat sequences were identified using blast N.
